# Supplementary material for: Cyanoexosortase B is essential for motility, biofilm formation, and scytonemin production in a filamentous cyanobacterium
Source: mSphere. 2025 May 13;10(6):e01006-24. doi: 10.1128/msphere.01006-24 (PMC12188702; doi:10.1128/msphere.01006-24)
Supplement: Supplemental materials — Table S1, Fig. S1-S4, Movie S1 and S2 legends, and Data Set S1 legend. [file msphere.01006-24-s0002.pdf]

Supplementary Materials for:

Cyanoexosortase B is essential for motility, biofilm formation and scytonemin production in a filamentous cyanobacterium

Gabriel A. Parrett[1], Daniel H. Haft[2], Maida Ruiz[3], Ferran Garcia-Pichel[3], Christopher C. Ebmeier[4], and Douglas D. Risser[1]*

1. Department of Biology, University of Colorado Colorado Springs, Colorado Springs, CO 80918 USA
2. National Center for Biotechnology Information, National Library of Medicine, National Institutes of Health, Bethesda, Maryland, 20892, USA
3. Center for Fundamental and Applied Microbiomics, Biodesign Institute, and School of Life Sciences, Arizona State University, Tempe, AZ 85281, USA
4. Proteomics and Mass Spectrometry Core Facility, Department of Biochemistry, University of Colorado at Boulder, Boulder, CO 80303

*To whom correspondence should be addressed. Email: drisser@uccs.edu

Table S1. Strains, Plasmids, and Primers

strain name	description	Source
N. punctiforme ATCC29133	wild type	ATCC
UOP217	Δ*crtB* (Npun_F0456)	This study
**plasmid name**	**description**	**Source**
pDDR539	Suicide vector for in-frame deletion of *crtB*	This study
pGAP100	Shuttle vector containing *crtB* expressed from the *petE* promoter	This study
**Primer name**	**sequence**	
NpF0456-5'-BamHI-F	atataggatccCAATTATTCCTGTACATCTCTATG	
NpF0456-5'-OEP-R	gttctaactttcGAGTGCCATGATTTTTATTGG	
NpF0456-3'-OEP-F	catggcactcGAAAGTTAGAACTATTGGGCATTG	
NpF0456-3'-SacI-R	atatagagctcGCTAAAGACCAAGATGTTTCTAC	
NpF0456-BamHI-F	atataggatccATGGCACTCCAGCAACAG	
NpF0456-SacI-R	atatagagctcCTAACTTTCTGTTTCTTGCTC	

SMOV 1. Time-lapse microscopy of wild-type and Δ*crtB* hormogonia.

SMOV 2. Time lapse microscopy of exogenous complementation of mutant strains by addition of culture medium containing HPS.

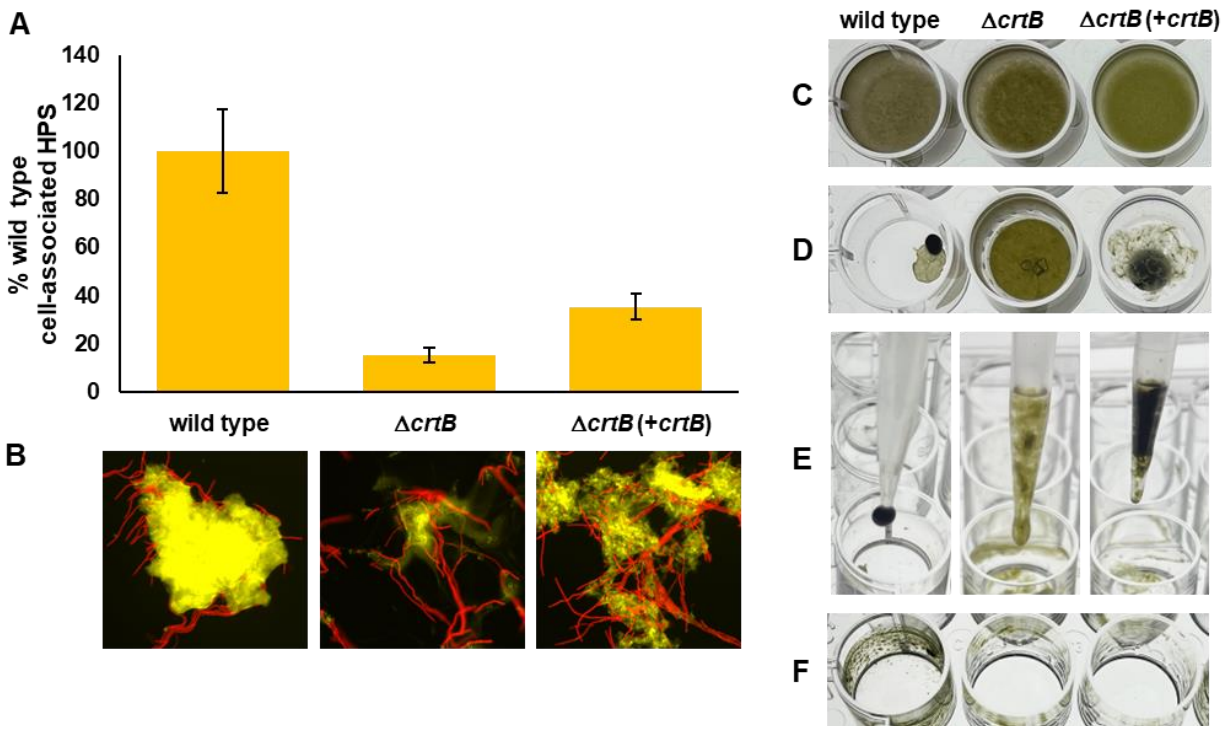

Figure S1. HPS accumulation, aggregation, and biofilm formation in the Δ*crtB* complementation strain. **(A)** Quantification of cell-associated HPS and **(B)** example fluorescence micrographs (red = autofluorescence, yellow = HPS) for the wild type, Δ*crtB*, and complemented Δ*crtB* strain (+*crtB*) as indicated. **(C-F)** Images of 2 mL cultures in 24 well plates demonstrating aggregation and biofilm formation for the wild type, Δ*crtB*, and complemented Δ*crtB* strain (+*crtB*) as indicated. **(C)** Cultures prior to hormogonium induction. **(D)** 24 h post hormogonium induction. The wild-type strain forms a dense aggregate and floating mat. The Δ*crtB* strain does not aggregate or form mats. The complemented strain forms an aggregate and mat, but these appear less dense. **(E)** Disruption of the 24 h post induction cultures by pipetting. The wild-type aggregate is dense enough to plug a pipette tip and the entire aggregate can be lifted out of the culture. The Δ*crtB* strain is easily disrupted by pipetting. The aggregate formed by the complemented strain is much looser than the wild type and more easily disrupted by pipetting. **(F)** Biofilms on the side wall of the culture vessels 5 days post induction, following the removal of cultures and washing of vessels. Only the wild type exhibits substantial biofilm accumulation.

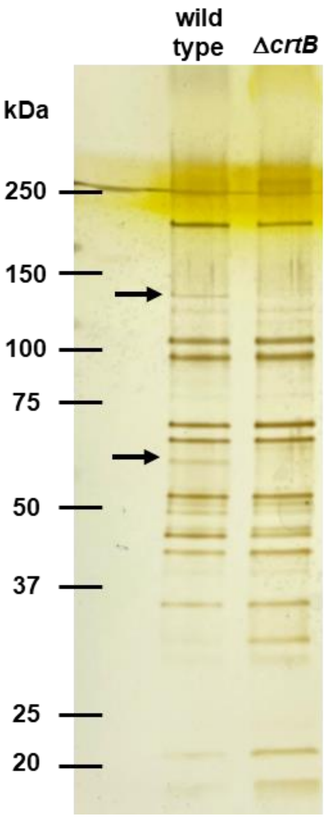

Figure S2. SDS-PAGE analysis of the hormogonium exoproteome. Concentrated cell-free culture supernatants were separated by SDS-PAGE and proteins detected by silver staining. Arrows indicate proteins detected in the wild type exoproteome that were absent or diminished in the Δ*crtB* proteome.

Data Set S1. Exoproteome analysis of the wild type and Δ*crtB* strains. (**A**) Parameter settings for Mass-spectrometry. (**B**) Summary of each sample and normalization. (**C**) proteinGroups with label free quantitation. (**D**) Results from limma analysis. (**E**) Results from limma analysis for PEP-CTERM proteins.

Npun_F4801
MSRADAASMTFSVTGTNSASNNALASSVVFDDLLNPGKLTVTLTNMKNVSVPSDVLTSVFWDYAGSPLNLSLISATAA
TVTKNNPSTTTNNVNLLNTPNGKEWAFASTTNSAGLTNGVTQDYGLGTAGLGIFQGIGGQQQVNYGIIDGYNANANS
PVKGGSFVDNSATFVLSGLPANFDIRKIGSVRFQYGTALSEPSIIKAQGNYYSPPPPPPKKVPEPGTTAALGLFAVGAL
KVVRKKSLVVA

Npun_R0434
MKTQSLFNLLKVGITTFGIFFVAGQGAYASDLKGTAFSISLECMNDTTGLLMGQNSTDANSWQYAFDSNKDGMNGNY
WVGAAPGKVNPYDISGMAIKETATSIIVAINGNMKLTGEAEQGAVGGQIGYGDLFFNMAGKTFDNAMSSGDLFGIHFA
SANASGVQQLGVYSGVQAKTVTNIKEGYTVATYGGLAGGGNSYESQVKQGGGIVGYGDLTSSYFTNNGKDNTFNLN
VIDSGKYLSGISFLAQGNVTQQLLTTGYDASKFNGTQTIAFEFKKSGVVQSTPEPASLAGLGIVGLALAGSKRRKKLA

Npun_R3960
MKMTFQKLVIGASMAIGVGAIATVPAQAGTLTGATIGGTAASDYLVYDMSGNSTVLVPNTQTNVQKVLDGNAANPTG
NVELRASSEQSGFDFSKNTTLTGQIGDKSITLSSLTATDWFSTGSVLSTSYGVNNFANTWFNQFYDAAGLASNESAIK
LALNLPSFTPSSFIRQQAFNAFYGIGGFQRSSDPNISYVNQNDTTGDIKIGLAGHYNLKDYYAPLLGSFGNFLKDGFQA
SEVVKVTYNGKTDFRYSFSATQSGLSNNSGPGADGKSHSGNYEVTIQGIPPAAVPEPSVMLGLLGVAGVFATQRKLK
KASI

Npun_ER021
MKNQNLFNLLKIGITTFGIFFVASQGAYASDLKGTAFSISLECMNDTTGLLMGQNSTDANGWQYAFDSNKDGMNGNY
WVGAAPGSVNPYDISGMAIKETATSIIVAINGNMKLTGEAEQGAAGGQIGYGDLFFNMAGKTFDNAMSSGDLFGIHFA
SANASGVQQLGVYSGVQAKTVTNIKEGYTVATYGGLAGGGNSYESQVKQGGGIVGYGDLTSSYFTNNGKDNTFNLN
VIDSGKYLSGISFLAQGNVTQQLLTTGYDASKFNGTQTIAFEFKKSGTVQSTPEPASLAGLGIVGLALAGSKRRKKSS

Npun_F0296
MKNLLIKSAIASGVVLSLATVSRPVMAASFAVSIENAGVQNAQLSNLVNAHVDTFDSETQGYSATGFQWNDGTKNIGS
YQNTLVMNAEQYGGAGGTGKYFDVDTNRSGNGQKVSTLNLATPQSYFGFWWSAGDSSNVLTFLSQGQVVYSMTT
ADVVNYIAKLPNKTSYYGNPNSTFKNQDSGEPFAFINFYDVGGTFDQVQFTNIGGTGFESDNHTVATGYNSINGNIVT
TAVPESSSLLGVFAIGFVGAASVMTRRVKKKSVLQLS

Npun_R6196
MIRFRKVSSIMTTKLLNTLAAATTLAGIVVTSGAANAASLSYSTSTNYEFTDIIDSPLSVQKFNSSLGTLQGITIEFTGDIL
GNAGFENRSPNARQATVNLGSQLSLKLNNQSLLELNPQYTSSYQVAKYDGISDYGGTSGKTLSNLTATQSGIQSFTD
TQLLQSFIGNGNVDFLFSAIADSEVKGSGNISSYIDTYAKAGIKLTYNYDIKSVPEPSATLGIGLIAGLCLLSQRKKSWLK
GSNS

Npun_R4127
MKVSSSGIKLGGAILSGSCLAAMTIALSGASAFAAQFITIESTGTLSGTIELPRNNPNFNNSITRIDTDDTGTYYRNLGTN
NNPNYVPVYQSDYLQVETRSDGSLHYFVDFKGIPFVSFDGVLTSPVLSGGQLTPYKYQGQLAGTKFQGVVQDEFNFI
KALYSGTVTDPDTGKQYQGTFEVSGYGVRYSDRNGNSTPTVFDFQSDIPGSPTVTSLNITNATLANLRIKVPIDETSIP
EPASVLGTFLVAGFGMVLKRKKHICSKKIF

Npun_F5844
MTTTVTKIALSLLGAAGISFLSLTPARAVILVEPIVTSVNEDIFNTKKPRILTDYEPGQVIEYGVPDVANNFLNNTGYDIES
LVFDLKTLSYTNPDSTPPFDNEPVEWGDVNGDGKIGLSNNPDLKDIFTDITISGSTITFSGGVIRNGSVFYNPFATLPNL
APGAGIIPPIPAEQADKDGPIRVAAYYTAVPESTNVLGVLVLGAVGVALKLKRSLHY

Npun_F1135
MSISTIAKKLSTAVIAATAIVLGIGTVAEATVLTFDDIAPISGYDRIPNGYGGFNWDNFGYLNGSDTALS
LTGYDNGSVSGDYVSFNEYGDPALVSDGIFDFNSAYLTAAWNDGLSVTVEGFKSGATLYSKTVVVDTKQPTLVNFDY
FGVDELRFTSFGGVEPDYLRSKGGPGTQFTLDNFTFNEKVTSVPEPTLLPAILSIATVSAGSV
LKRKQLKQS

Npun_DR029

MKFFNFCQIGVPLISFALLGWIAPRANAALLVGSYNNNSVLRYEEKTGEFIDEFVPNGSGGLQGTTGLTIGPDNNLYVS
SILNGSIQRYDGKTGNFISTFVPSGSGGLDAPEDLVFGPDGNLYVTSINSRTSNSVLRYNGQTGAFIDTFVPSSSGGLT
APFGLSFGSDNNLYVSSVLTDNILRYNGQTGAFIDTFATEAKGSEPGGLTFGADGNLYVANFVSNNISRFNGKTGAFID
TFIPAGSGGLNDPLKPVFGPDGNLYVSGLNSNNVLRYDGKTGAFIDTFIPAGRGGLNGAGFLAFTEDNTIIPEPRMTLA
VLAFGATLGASKLLSRKQKSKILLG

Npun_R1271
MGLVKNLSIGILGTGFIVLTTAAQAKAVTLSYDRSIGSPGFGPGELFVPQGITVDSQGNTLIANGRGVNPDGTPNYNVG
NKIEKFSPSGQYIGAIGSGGTGPGQFDEPTTVDFNPVTGDLYAGDVYNNRINQFDSQGNFIRSFANGEFTPLVEGRLF
FGPSGVTFDKTGNVYIGDFNGERILKFTPDGQQIGVIGGTLGTAPGQFQGVAGLRISPVSGNIFVADQYNNRVQVLDP
NGNPLLAFGSAGSGPGQLLQPIGIEVDDQENVYVADSINSRVQVFDKNGNFLTSFGENARDASGNPVPPPALTGPPF
GNPLDLTPGRFNWTGGTSYKDGKLYVGDFFQGRVQVINVEGRKQVPEPGSALGLALLGLGAATITLRKRGQQKPVFS
LEKELQKQC

Npun_F5792
MISLKSKLLNATLVATAALPLAVAGLFTSAGSAQAAALKGEFSFNGGLTQNPFASSLVTLKKDSLTFTPDSVIADANGV
KSTPVAIAAQTGSFLGFNSASIRDIISFTGNTVQNPFLDLGNLTLPGIVLNGESTASLADGSNIFTLTSSGYKITQTGANIT
IAVDLFGYFTSATGEITKGAGSLSFQRNDTTVAQANAILNTTGGSLANLTFSGSTFTTSVPEPAALLGLGTVGAVMVMS
RRRKSVV

Figure S3. Peptide coverage for PEP-CTERM proteins detected in the exoproteome. Green = predicted general secretory pathway signal peptide. Red = PEP-CTERM domain. Yellow highlight = peptide detected by mass-spectrometry.

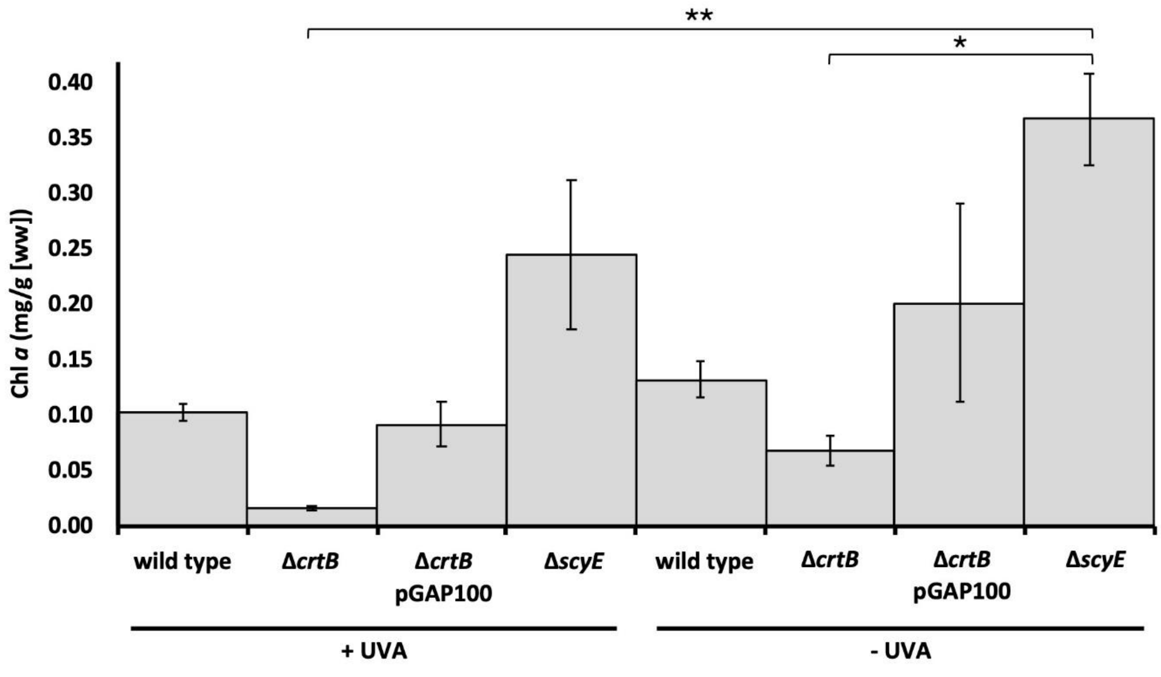

Figure S4. Chlorophyll *a* concentration in strains as indicated in the presence and absence of UVA exposure. * = $p<0.05$, ** = $p<0.01$ based on Nemenyi post-hoc tests.
